# A Model of Superinfection of Virus-Infected Zebrafish Larvae: Increased Susceptibility to Bacteria Associated With Neutrophil Death

**DOI:** 10.3389/fimmu.2018.01084

**Published:** 2018-05-24

**Authors:** Laurent Boucontet, Gabriella Passoni, Valéry Thiry, Ludovico Maggi, Philippe Herbomel, Jean-Pierre Levraud, Emma Colucci-Guyon

**Affiliations:** ^1^Institut Pasteur, Unité Macrophages et Développement de l’Immunité, Paris, France; ^2^CNRS UMR 3738, Paris, France

**Keywords:** Sindbis virus, *Shigella flexneri*, co-infection, zebrafish, neutrophils, live imaging, innate immune response

## Abstract

Enhanced susceptibility to bacterial infection in the days following an acute virus infection such as flu is a major clinical problem. Mouse models have provided major advances in understanding viral-bacterial superinfections, yet interactions of the anti-viral and anti-bacterial responses remain elusive. Here, we have exploited the transparency of zebrafish to study how viral infections can pave the way for bacterial co-infections. We have set up a zebrafish model of sequential viral and bacterial infection, using sublethal doses of Sindbis virus and *Shigella flexneri* bacteria. This virus induces a strong type I interferons (IFN) response, while the bacterium induces a strong IL1β and TNFα-mediated inflammatory response. We found that virus-infected zebrafish larvae showed an increased susceptibility to bacterial infection. This resulted in the death with concomitant higher bacterial burden of the co-infected fish compared to the ones infected with bacteria only. By contrast, infecting with bacteria first and virus second did not lead to increased mortality or microbial burden. By high-resolution live imaging, we showed that neutrophil survival was impaired in Sindbis-then-*Shigella* co-infected fish. The two types of cytokine responses were strongly induced in co-infected fish. In addition to type I IFN, expression of the anti-inflammatory cytokine IL10 was induced by viral infection before bacterial superinfection. Collectively, these observations suggest the zebrafish larva as a useful animal model to address mechanisms underlying increased bacterial susceptibility upon viral infection.

## Introduction

Despite steady progress in their diagnosis and treatment, viral and bacterial diseases continue to spread across the world and are causing a huge societal burden in terms of health and economical costs. Frequently, viruses and bacteria infect the same host, resulting in more severe illness compared to single infections. The best example is influenza-associated bacterial pneumonia, where bacterial superinfections have been documented as the major cause of death in major influenza pandemics but also during seasonal influenza epidemics (1, 2). *Staphylococcus aureus, Streptococcus pneumoniae*, and other bacteria commonly found even in healthy people have been identified to be associated in co-infections with influenza virus, causing severe and lethal pneumonias in influenza virus-infected people. Other common viruses (e.g., rhinoviruses, enteroviruses, or rotaviruses) have been also associated in co-infection and have been reported causing increased susceptibility to a variety of bacteria (3). In general, polymicrobial infections result in a synergy among the various microbes, increasing the susceptibility and interfering with the immune response (4). To our knowledge, the only well-documented cases of increased resistance of the host to a second microbe, after a first infection with another microbe, occur after usage of vaccinal strains resulting in “trained immunity” (5).

Mammalian animal models are extensively used to address the mechanisms of increased bacterial susceptibility upon viral infection, using mostly influenza virus and bacteria found to be associated with co-infection during influenza episodes in humans (6). It has been shown that viruses could damage lung epithelium, favoring bacterial attachment and invasion (7, 8). However, immune interference is generally believed to be a more important factor than tissue damage (9). Several possible immunological mechanisms have been studied. Anti-bacterial and anti-viral innate immune responses are different as they involve the induction of largely distinct cytokines and signaling pathways, which could interfere with each other (10). Clear impact on myeloid cell recruitment or survival has been reported, but sometimes with conflicting results (11–15). Perturbed cytokine and chemokine induction (16, 17), as well as impaired bacterial killing (18, 19), have also been reported. Third, the crucial role of type I interferons (IFN) in modulating these aspects of antibacterial immunity has been demonstrated using IFNAR knockout mice models, that have been shown more resistant to bacterial superinfection (11, 12). However, modeling the anti-viral immune response by injecting recombinant IFNs has also shown that IFN alone does not fully recapitulate the increased bacterial susceptibility upon viral infection (16). Thus, although mouse models have provided major advances in understanding viral–bacterial superinfections, the mechanisms of this hyper-susceptibility remain an intense area of investigation.

The zebrafish (*Danio rerio*) has become a valuable non-mammalian vertebrate model to study infectious diseases. The zebrafish larva is an excellent system for live imaging, being transparent, small, and easy to anesthetize. With the availability of transgenic lines harboring fluorescent leukocytes coupled with diverse tools to manipulate immune cells and pathways, it offers the unique opportunity to study the immune response and leukocyte behavior *in vivo* upon infection in an entire vertebrate organism. As a vertebrate, it shares immune cell types and pathways with mammals, and it has been successfully used to study host-pathogen interactions using a variety of microbes causing disease in humans, including bacteria, fungi, and viruses (20–22).

Sindbis virus (SINV), the prototype species of the *Alphavirus* genus (positive strand RNA virus), is widely used as an experimental model in mice and can infect a broad range of vertebrates and insect cells (23). We recently established an infection model of this enveloped, single-stranded positive RNA virus in zebrafish (24). SINV infection in zebrafish is highly similar to that caused by its relative chikungunya virus (CHIKV) (25); both viruses replicate rapidly during the first day, then the viral burden stabilizes, correlating with the induction of a strong type I IFN response. Virus-infected cells visualized thanks to a GFP reporter in the virus genome, and present in many organs, then disappear progressively from most of the body, although infection persists longer in the central nervous system. CHIKV infection in zebrafish larvae causes an increase of the number of neutrophils, which are a major source of type I IFN. While zebrafish larvae normally survive CHIKV infection, lethality ensues after IFN receptors knockdown (25).

*Shigella flexneri* (hereafter simply designated as *Shigella*) are human-adapted Gram negative bacteria, close relatives of *Escherichia coli* that have gained the ability to invade the colonic mucosa, causing inflammation and diarrhea (26). We have previously established that *Shigella* is pathogenic for zebrafish larvae. We have shown that zebrafish survival is dose-dependent upon *Shigella* injection, where sublethal doses are cleared within 48 h post infection, and lethal doses causing the death of the infected larvae with concomitant high bacterial burden. Although both macrophages and neutrophil engulf the injected *Shigella*, we have highlighted a scavenger role for neutrophils in eliminating infected macrophages and non-immune cell types that have failed to control *Shigella* infection, thus playing a crucial role in anti-*Shigella* defense. However, both macrophages and neutrophils undergo cell death in larvae injected with high lethal *Shigella* inocula, and leukocyte depletion is associated with bacteremia preceding the death of the larvae (27).

The aim of this work was to test whether the zebrafish larva could be used to model viral-bacterial co-infections *in vivo*. Here, we have set up a zebrafish model of sequential viral and bacterial infection, using sublethal doses of SINV and *Shigella*. We have shown that larvae infected with SINV first display an increased susceptibility to *Shigella* infection, associated with death of the co-infected fish and increased bacterial burden. By contrast, larvae infected with *Shigella* first and SINV second do not show any difference in survival and pathogens dissemination compared to single viral and bacterial infection. We also observed that neutrophils, key players in anti *Shigella* defense, were severely depleted upon SINV + *Shigella* co-infection. By high-resolution live imaging, we documented the death of bacteria-engulfing neutrophils. We also measured the induction of the main cytokines genes. Co-infection did not blunt expression of typical antibacterial cytokines (*il1b* and *tnfa*); however, both type I IFN and the anti-inflammatory cytokine *il10* were induced by the viral infection prior to bacterial superinfection, suggesting a contribution to the observed phenotype.

These observations highlight the zebrafish model to study how viral infections can pave the way for bacterial co-infections. Moreover, this model could offer the opportunity to screen, in a live organism, libraries of anti-microbial, or immuno-modulating compounds.

## Materials and Methods

### Ethic Statement

Animal experiments were performed according to European Union guidelines for handling of laboratory animals (http://ec.europa.eu/environment/chemicals/lab_animals/home_en.htm) and were approved by the Institut Pasteur Animal Care and Use Committee.

### Zebrafish Care and Maintenance

Wild-type AB fish, initially obtained from the Zebrafish International Resource Center (Eugene, OR, USA) and Tg(*mpx:GFP)^i114^* (28), were raised in our facility. Eggs were obtained by marble-induced spawning, bleached according to standard protocols, and then kept in Petri dishes containing Volvic source water and, from 24 hours post fertilization (hpf) onward 0.003% 1-phenyl-2-thiourea (PTU) (Sigma-Aldrich) was added to prevent pigmentation. Embryos were reared at 28°C or 24°C according to the desired speed of development; infected larvae were always kept at 28°C. All timings in the text refer to the developmental stage at the reference temperature of 28.5°C (29). Larvae were anesthetized with 200 µg/ml tricaine (Sigma-Aldrich) during the injection procedure as well as during *in vivo* imaging.

### Viruses

Sindbis viruses were produced on BHK cells [originally obtained from American Type Culture Collection (ATCC), #CC-L10], according to Ref. (30). Two SINV-GFP strains were used, both based on the hybrid TE12 strain backbone. SINV-3′GFP, the strain previously tested in zebrafish in Ref. (24), harbors a 3′ genomic insertion of the eGFP gene under the control of a second subgenomic promoter (31). The SINV-eGFP/2A harbors a self-cleavable eGFP inserted between the capsid and pE2 regions, based on (32). Briefly, the pTE-3′2 J GFP4-10 plasmid, which encodes for the SINV-3′GFP genome, was first modified to replace the region downstream of the structural genes (including the second subgenomic promoter and eGFP) with the 3′UTR from the AR339 strain. This region was amplified by PCR using pTR339-mCherry2A (Sun et al., 2014) as a template with primers SINV-E1-end-F (GACTAGCACACGAAGATGAc) and SINvec_Xho-R (AATTCCCCTCGAGGAATTCC), while PTE-3′2 J GFP4-10 was digested by ApaI and XhoI; purified fragments were then reassembled using In-Fusion^®^ HD Cloning Kit Clontech/Takara (#639650), and after transformation in *E. coli*, plasmid pTE3′2J-3′UTR-339 was obtained. The eGFP-2A fragment, and some flanking regions (identical in TE12 and AR339 SINV strains) was then amplified by PCR from pTR339-EGFP2A using primers SINV-C-pml-F (GGTAATGAAACCTCTGcacg) and SIN-E3-stu-R (ATTGAGCAGGGTATCGTagg), and was then subcloned into pTE3′2J-3′UTR339 digested by PmlI and Stu I enzyme. This yielded the pTE3′2J-eGFP2A-3′UTR339, which was verified by sequencing and then used to produce the SINV-eGFP2A virus.

### Virus Titration

Virus titer from concentrated BHK supernatants was measured on Vero-E6 cells (ATCC #CRL-1586) as described in Ref. (24). In addition, the infectivity of the virus in zebrafish cells was also measured by microinjection of serially diluted virus suspensions in the cell mass of dome stage AB zebrafish embryos), followed by observation of GFP expression one day later; the two methods yielded consistent titers.

### Bacteria

Bacterial strains used in this study were wild-type invasive of *Shigella flexneri* serotype 5a M90T expressing DsRed (33). *Shigella* were plated from −80°C glycerol stock onto a Congo Red tryptic casein soy agar plate; a virulent clone was cultured overnight in trypticase soy complemented with ampicillin (50 µg/ml), and then diluted 80× in fresh trypticase soy, and cultured until *A*_600nm_ = 0.6. The bacterial exponential subculture was centrifuged at 1,000 × *g* for 5 min and the pellet washed with PBS and centrifuged at 1,000 × *g* for 5 min. The pellet was reconstituted with 60 µl of PBS for inoculation.

### Zebrafish Infections

Titered viral suspensions were stored at −80°C as 10 µl aliquots, and one aliquot was used per experiment. The volume of injected suspension was deduced from the diameter of the drop obtained after mock microinjection, as described in Ref. (34); typically, ~3 nl of a 2.10^7^ PFU/ml suspension was injected intravenously (iv) for a 60 PFU inoculum. Bacteria were recovered by centrifugation, washed, resuspended at the desired concentration in PBS. 72 or 96 hpf anesthetized zebrafish larvae were microinjected iv with 0.5–2 nl of bacterial suspension as described previously (27). Local bacterial infections were performed by injecting subcutaneously 0.5–1 nl of bacterial suspension to 96 hpf zebrafish larvae as previously described (35). The exact inoculum was checked *a posteriori* by injection in a water drop and plating onto LB agar. Infected larvae were transferred into individual wells (containing 1 ml of Volvic water + 0.003% PTU in 24-well culture plates), incubated at 28°C and regularly observed under a stereomicroscope.

### Morpholino Injections

Morpholino antisense oligonucleotides (Gene Tools) were injected at the one to two cells stage as described (32). crfb1 splice morpholino (2 ng, CGCCAAGATCATACCTGTAAAGTAA) was injected together with crfb2 splice morpholino (2 ng, CTATGAATCCTCACCTAGGGTAAAC), knocking down all type I IFN receptors (23). Control morphants were injected with 4 ng control morpholino, with no known target (GAAAGCATGGCATCTGGATCATCGA).

### Measurement of Bacterial Burden

At the indicated times, animals were anesthetized, rinsed, and collected in 150 µl of sterile water. The animals were lysed and homogenized with a polypropylene piston (ten up-and-down sequences). Four serial 10-fold dilutions of the homogenates were plated onto LB agar, and CFU were enumerated after 24 h of incubation at 37°C; only colonies with the appropriate morphology and color were scored.

### Live Imaging, Image Processing, and Analysis

Quantification of total neutrophils numbers on living transgenic reporter larvae was performed upon viral and bacterial infections as we previously described (27). Briefly, bright field, DsRed, and GFP images of whole living anesthetized larvae were taken using a Leica Macrofluo™ Z16 APOA (zoom 16:1) equipped with a Leica PlanApo 2.0X lens, and a Photometrics^®^ CoolSNAP™ *HQ*2 camera. Images were captured using the Metavue software version 7.5.6.0 (MDS Analytical Technologies). Using these settings, it was possible to discriminate between the GFP from the SINV-GFP infected cells (diffuse and weak) and the GFP signal from neutrophils (concentrated and bright). After capture of images, larvae were washed and transferred in a new 24-well plate filled with 1 ml of fresh water in each well, incubated at 28°C and imaged again under the same conditions the day after.

Then pictures were analyzed and neutrophils (*mpx*:GFP + bright cells) were manually counted using the ImageJ software version 10.2 (developed by the National Institute of Health). Counts shown in Figures 3B and 4B are numbers of neutrophils per image.

High resolution confocal live imaging of infected larvae was performed as previously described (27, 35, 36). Briefly, the injected larvae were positioned in 35 mm glass-bottom dishes (Inagaki-Iwaki) and immobilized in the dish with a 1% low-melting-point agarose and then covered with 2 ml Volvic water containing tricaine. Confocal microscopy was performed at 23–26°C. A Leica SP8 confocal microscope equipped with two PMT and Hybrid detector, a 20X oil immersion objective (HC PL APO CS2 20X/0.75) and a X–Y motorized stage was used to live image SINV + *Shigella* co-infected and *Shigella* only infected larvae (represented in Figure 4C; Video S1 in Supplementary Material). To simultaneously acquire SINV + *Shigella* and *Shigella* infected larvae, the “mark and find” mode of acquisition was applied. A Leica SPE inverted microscope and a 40 × oil immersion objective (ACS APO 40 × 1.15 UV) was also used to live image SINV + *Shigella* co-infected larvae represented in Figure 4F and Video S2 in Supplementary Material. The 4D files generated by the time-lapse acquisitions were processed, cropped, analyzed, and annotated using the LAS-AF Leica software. Acquired Z-stacks were projected using maximum intensity projection and exported as AVI files. Frames were captured from the AVI files and handled with Photoshop software to mount figures. AVI files were also cropped and annotated with ImageJ software, then compressed and converted into QuickTime movies with the QuickTime Pro software. Neutrophils were manually counted and tracked over time from maximum intensity projection movies of infected larvae.

### Cytokine Expression, Viral and Bacterial Burden Measurement by qRT-PCR

RNA was extracted from individual larvae using RNeasy^®^ Mini Kit (Qiagen). cDNA was obtained using M-MLV H- reverse-transcriptase (Promega) with a dT_17_ primer or a random nonamer (for host and bacterial transcripts, respectively). Quantitative PCR was then performed on an ABI7300 thermocycler (Applied Biosystems) using Takyon™ ROX SYBR^®^ 2× MasterMix (Eurogentec) in a final volume of 25 µl. The following pairs of primers were used:

*ef1a* (housekeeping gene used for normalization): GCTGATCGTTGGAGTCAACA and ACAGACTTGACCTCAGTGGT
*ifnphi1* (secreted isoform): TGAGAACTCAAATGTGGACCT and GTCCTCCACCTTTGACTTGT
*il1b*: GAGACAGACGGTGCTGTTTA and GTAAGACGGCACTGAATCCA
*il10*: CATAACATAAACAGTCCCTATG and GTACCTCTTGCATTTCACCA
*tnfa*: TTCACGCTCCATAAGACCCA and CAGAGTTGTATCCACCTGTTA
*mmp9*: AACCACCGCAGACTATGACAAGGA and GTGCTTCATTGCTGTTCCCGTCAA
*E1-SINV*: GACAACATGCAATGCAGAATG and CTAGTCAGCATCATGCTGCA
*il22*: TGCAGAATCACTGTAAACACGA and CTCCCCGATTGCTTTGTTAC
*cxcl8a*: GTCGCTGCATTGAAACAGAAAGCC and CTTAACCCATGGAGCAGAGGGG
*il23a*: CTGAAAGTGCTTAAGGAATCGG and GAGAAGGAGTAGAGTCTTTCCAC
*ifng1r*: ACCAGCTGAATTCTAAGCCAA and TTTTCGCCTTGACTGAGTGAA
*dram1*: CCTGGTTATCTGGTCATCGA and CATGAATCCAAACACACAGCT
*DsRed*: CAAGGAGTTCATGCGCTTC and TACATCCGCTCGGTGGA

### Statistical Analysis

Normal distributions were always analyzed with the Kolmogorov–Smirnov and the Shapiro–Wilk tests. To evaluate difference between means of normally distributed data (for neutrophil numbers and bacterial burdens) (Figures 2D, 3B, 4B, 4D and 4F; Figures S2C,D in Supplementary Material), an analysis of variance followed by Bonferroni’s multiple comparison tests was used. For bacterial burdens (CFU counts), values were Log10 transformed. For cytokines expression and some bacterial burdens (Figures 2C and 5; Figures S3–S5 in Supplementary Material), non-Gaussian data were analyzed with the Kruskal–Wallis test followed by Dunn’s multiple comparison test. *P* < 0.05 was considered statistically significant (symbols: ***P < 0.001; **P < 0.01; *P < 0.05). Survival data were plotted using the Kaplan–Meier estimator and log-rank (Mantel–Cox) tests were performed to assess differences between groups. Statistical analyses were performed using GraphPad Prism^®^ software.

## Results

### Establishing Zebrafish as a Model for Viral Bacterial Co-Infections

**Figure 1:**
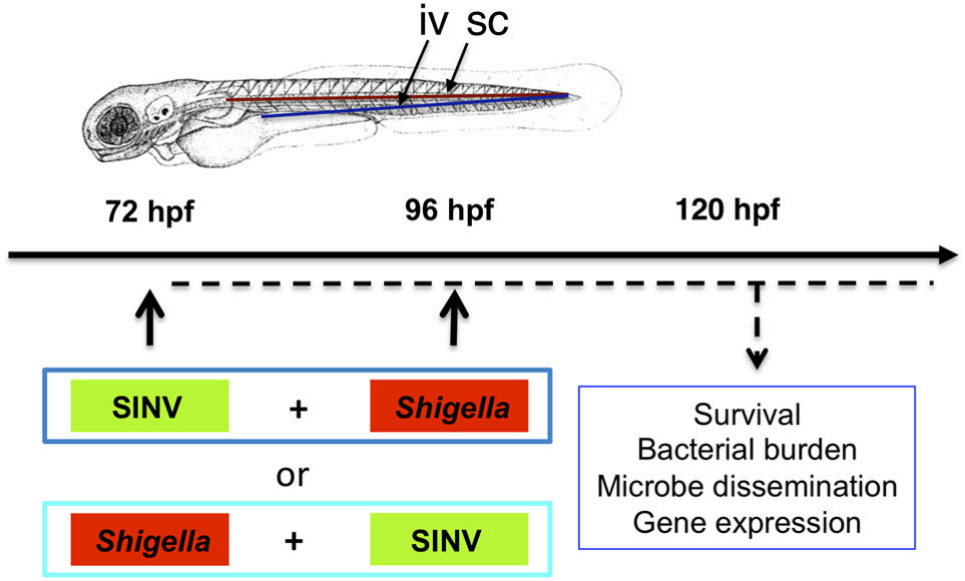
Modeling viral-bacterial co-infection in zebrafish. Scheme of the experimental set up of viral bacterial co-infection using zebrafish. A 72 hpf zebrafish larva is shown. Microbes are injected in the bloodstream [intravenously (iv)] *via* the dorsal aorta (red line) or the ventral vein (blue line). Subcutaneous injections of the bacteria (sc) are performed over a somite, in the caudal region of the larva. Sindbis virus (SINV) and *Shigella flexneri* bacteria (*Shigella*) are sequentially injected in the bloodstream (iv) of zebrafish larvae at 72 and 96 hpf. Both SINV + *Shigella* and *Shigella* + SINV sequential co-infections are tested. Non-injected fish and fish injected with SINV or *Shigella* alone are used as a control. Single SINV or *Shigella* injections are performed at 72 or 96 hpf depending on the sequential co-infection tested. Survival, viral replication, bacterial burden, neutrophil behavior and expression of antiviral and antibacterial related genes are monitored over time as represented (dotted black line).

To test whether the zebrafish larva could be a valuable model to address mechanisms of viral and bacterial co-infection *in vivo*, we decided to combine our well-characterized SINV and *Shigella* zebrafish infection models (Figure 1).

We used SINV-GFP viruses derived from the TE12 strain, which is moderately virulent in zebrafish (24). Unlike the strain used in our previous study (SINV-3′GFP), which bears an eGFP sequence in the 3′ region of the region preceded by an additional subgenomic promoter (31), the SINV-GFP2A virus bears an eGFP gene inserted in the structural ORF with a self-cleaving 2 A linker, thus being less prone to GFP loss upon replication (32). Both viruses led to a comparable amount of GFP signal in infected cells. Injecting 50–100 PFU of either virus into the bloodstream of 72 hpf zebrafish larvae results in infection of various cell types, easily observed thanks to the GFP gene inserted in the viral genome. The infection, however, remains sublethal, as viral burden quickly stabilizes after one day of rapid viral replication (24). However, upon type I IFN receptor knockdown, SINV infection is lethal (Figure S1 in Supplementary Material), showing that type IFN I plays a key protective role against SINV, similar to what has been described for the closely related CHIKV (25).

The M90T strain of *Shigella* causes a dose-dependent disease after inoculation to 72 hpf zebrafish larvae. An inoculum of up to 2,000 CFU is non-lethal and bacteria will be cleared in ~3 days, with phagocytes, and particularly neutrophils, playing a crucial role, while higher doses result in unbridled bacterial proliferation associated with macrophage and neutrophil depletion (27).

We combined SINV-GFP with *Shigella*-DsRed, allowing us to simultaneously monitor the dissemination of the two microbes. In the design of the sequential co-infection of zebrafish with virus and bacteria, we decided to inject one pathogen at 72 hpf, and the other 24 h later, when a robust response to the first one is established. We tested both SINV + *Shigella* or *Shigella* + SINV co-infection; single injections were used as controls. The scheme of the experimental set-up we designed is represented in Figure 1.

### Increased Susceptibility to Co-Infection Is Only Observed When Virus Is Injected First

**Figure 2:**
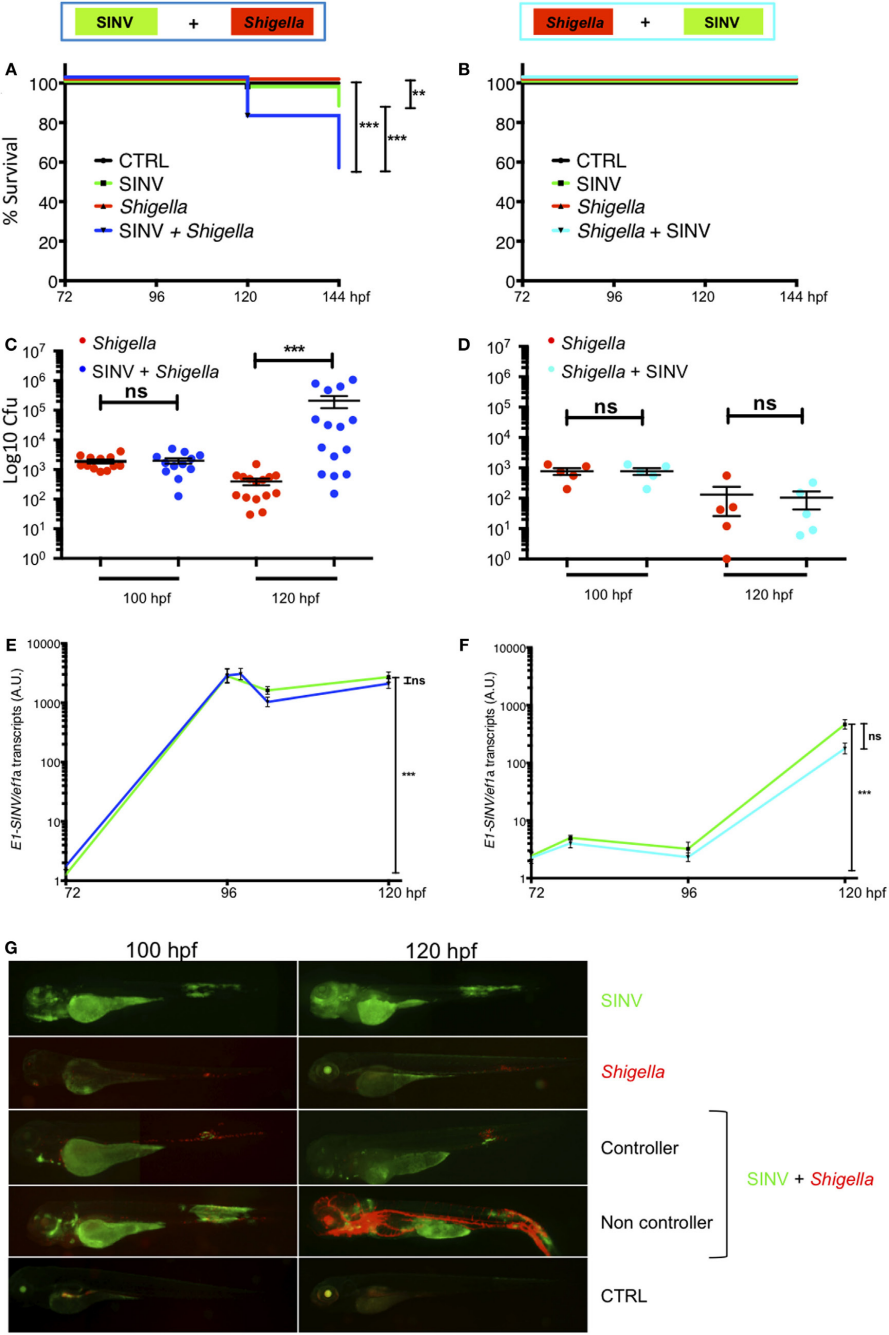
Increased susceptibility to co-infection is only observed when virus is injected first. Zebrafish larvae were sequentially injected in the bloodstream with Sindbis virus (SINV) and *Shigella* at 72 and 96 hpf and larvae injected with SINV or *Shigella* alone were used as a control as depicted in Figure 1. Survival, bacterial burden, and viral replication were evaluated over time in SINV + *Shigella* **(A,C,E)** and in *Shigella* + SINV **(B,D,E)** sequentially infected larvae settings. **(A,C,E)** Data pooled from three independent experiments; **(B,D,E)** data from one experiment; see also Figures S2A,B in Supplementary Material. Survival curves of zebrafish larvae injected with SINV + *Shigella* **(A)** (blue curve) or with *Shigella* + SINV **(B)** (cyan curve) and incubated at 28°C. For both sequential co-infection settings, fish injected with *Shigella* (red curves) or with SINV (green curves) alone at the appropriate time point, and non-injected fish (black curves) were used as controls. *n* = 72 **(A)** or 24 **(B)** fish for each condition. **(C,D)** Bacterial burden quantification by enumerating live bacteria in homogenates from individual larvae sequentially co-infected with SINV + *Shigella* **(C)** (blue symbols) or *Shigella* + SINV **(D)** (cyan symbols) or with *Shigella* alone (red symbols) measured by plating onto LB immediately after *Shigella* injection and 24 h post *Shigella* injection. *n* = 15 **(C)** or 5 **(D)** larvae for each condition. **(E,F)**. Viral replication measured by RT-qPCR from individual infected larvae in SINV-*Shigella* **(E)** (blue curve) or *Shigella*-SINV **(F)** (cyan curve) sequentially co-infected fish, or SINV (green curves). *n* = 15 **(E)** or 5 **(F)** larvae for each condition. **(G)** Representative images of virus (SINV-GFP) and bacteria (*Shigella*-DsRed) dissemination, determined by live imaging using a fluorescence stereomicroscope, of zebrafish larvae infected with SINV-GFP alone at 72 hpf, or with *Shigella* DsRed alone at 96 hpf, or sequentially co-infected with SINV-GFP first and *Shigella*-DsRed 1 day later. Non-infected larvae (CTRL) are also shown. The same infected larvae were live imaged 4 and 24 h post *Shigella* injection. Overlay of GFP and DsRed fluorescence is shown, except in SINV panels, where only GFP fluorescence was recorded.

We tested the outcome of a sublethal (~60 PFU) SINV-GFP2A inoculation at 72 hpf followed by a sublethal (~2,000 CFU) *Shigella*-DsRed injection at 96 hpf (SINV + *Shigella*, Figure 2A), as well as the opposite combination, namely injecting *Shigella* at 72 hpf, followed the SINV injection at 96 hpf (*Shigella* + SINV, Figure 2B). We then assessed the survival of the infected fish at 28°C by regular observation using a stereomicroscope. As expected with these sublethal doses, single injections of bacteria or virus, performed at either 72 or 96 hpf, did not result in mortality; upon termination of the experiment at 144 hpf, these animals did not display any overt signs of disease. *Shigella* + SINV co-infection did not result in significant mortality (Figure 2B). In striking contrast, when *Shigella* was injected after SINV, we recorded the death of about 50% of the co-infected fish within 2 days (Figure 2A). This result was reproducibly observed in three independent experiments and was also observed with the SINV-3′GFP strain (Figure S2 in Supplementary Material).

We then assessed pathogen burden in co-infected larvae. Viable bacteria were quantified by plating serial dilution of homogenates of euthanized fish onto bacterial culture dishes over time. As shown in Figures 2C,D, survival of *Shigella*-infected larvae (either single-infected or *Shigella* + SINV sequentially infected) was associated with clearance of bacteria over time. In contrast, in larvae inoculated with SINV first followed by *Shigella* injection (SINV + *Shigella*), *Shigella* numbers dramatically increased during the first 24 h post *Shigella* injection in about half the larvae (Figure 2C). This fraction, consistent with the 50% survival rate observed previously (Figure 2A), suggested that these larvae were unable to restrict *Shigella* proliferation and succumbed to the bacterial infection. By contrast, when we measured SINV transcripts by qRT-PCR, we found that SINV replication was essentially unaffected by either previous or subsequent *Shigella* co-infection (Figures 2E,F).

In addition, we observed the co-infected fish under the fluorescence stereomicroscope to assess the distribution of virus-infected cells and of bacteria, revealed by green and red fluorescence, respectively. This immediately confirmed that SINV + *Shigella*-infected larvae displayed bacterial, but not viral, overgrowth (Figure 2G). By contrast, imaging of the reciprocally co-infected larvae did not suggest any interference of the two infections (not shown). In double- or single-infected larvae, SINV distribution patterns were similar, with frequent infection of the large yolk cell, and of many cells in the jaw, of muscle fibers close to the injection zone in the tail, with subsequent propagation to the spinal cord and/or the brain, as previously described (24). In all *Shigella*-infected animals, 4 h after *Shigella* injection (100 hpf), the fluorescent bacteria were visible as specks mostly localized in the caudal hematopoietic tissue as well as in the vein over the yolk, consistent with rapid capture of bacteria by blood-exposed phagocytes which are abundant in these areas (35), and as previously described (27). However, 1 day later, the infection course was radically different between the *Shigella*-only and the SINV + *Shigella*-infected animals. At this time point, larvae that received *Shigella* only had almost all cleared the infection, showing few foci of *Shigella* mainly located near the injection point in the caudal part of the larvae. In contrast, half of the SINV + *Shigella*-infected larvae showed an uncontrolled *Shigella* proliferation, with dissemination in the bloodstream (bacteremia) and in the tissues near the site of injection. By daily observation of individual co-infected larvae under the fluorescent microscope to monitor bacterial dissemination, we observed that SINV + *Shigella* co-infected larvae that had controlled the bacterial proliferation at 120 hpf as suggested by the decreased level of bacterial fluorescence (e.g., 24 h post *Shigella* inoculation) usually survived; by contrast, SINV + *Shigella* co-infected larvae that exhibited high level of bacterial fluorescence at 120 hpf, usually were unable to control *Shigella* proliferation and died between 120 and 144 hpf (e.g., 24 and 48 h post *Shigella* inoculation). (*n* = 24 larvae scored for each condition; 100% survival for control and single infected larvae; 54% survival of SINV + *Shigella* co-infected larvae, 6/24 died at 120 hpf and 7/24 died at 144 hpf. All dead larvae were full of fluorescent bacteria.) This implies a critical time window early after *Shigella* inoculation.

Collectively, these observations show that SINV-infected zebrafish have an increased susceptibility to subsequent *Shigella* co-infection, establishing the zebrafish as a suitable model for the study of virus-induced hyper-susceptibility to bacterial superinfection.

### Impaired Neutrophil Counts Upon *Shigella* Injection in SINV Infected Fish

In mammals, professional phagocytes play key roles in containing *Shigella* infection, especially neutrophils that efficiently kill the bacteria they engulf (37), while macrophages (but not monocytes) actually get invaded (38). Similarly, in zebrafish larvae, we have previously shown that professional phagocytes contain the bacteria immediately upon the injection; furthermore, if *Shigella* may persist and replicate inside macrophages, neutrophils that have engulfed similar amounts of bacteria efficiently kill them. Neutrophils play an essential scavenging role by immediately engulfing debris and bacteria released by dying infected macrophages on non-immune cells, thus preventing bacterial dissemination (27). Considering this crucial role of neutrophils, we addressed their status in our SINV + *Shigella* co-infection model.

**Figure 3:**
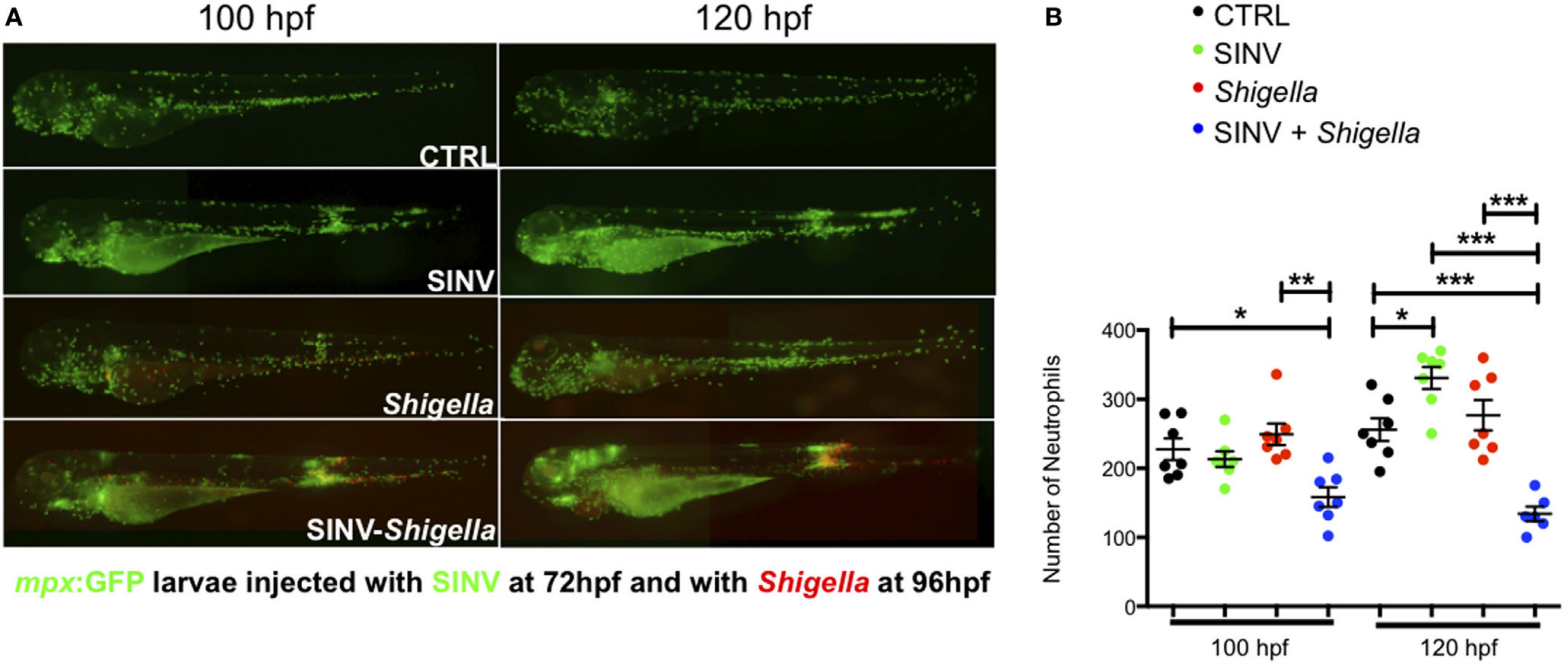
Impaired neutrophil counts upon *Shigella* injection in Sindbis virus (SINV) infected fish. **(A)** *mpx*:GFP zebrafish larvae (green neutrophils) were sequentially injected intravenously with sublethal doses of SINV-GFP at 72 hpf and the day after with sublethal doses of *Shigella*-DsRed. As a control, similar doses of SINV-GFP only and *Shigella* DsRed only were injected in the blood of 72 and 96 hpf *mpx*:GFP larvae, respectively. The infected larvae were then imaged with a wild field fluorescent microscope over time at 100 hpf (4 h post *Shigella* injection) and at 120 hpf (24 h post *Shigella* injection) to monitor the impact of the co-infection on the neutrophil population at the level of the entire organism. Note that it was possible to discriminate under the microscope the GFP label of the SINV-GFP infected cells (diffuse and less bright) from the GFP label of the *mpx*:GFP neutrophils (brighter). The neutrophil numbers appeared to be decreased in SINV-*Shigella* co-infected larvae. Overlay of green (SINV and neutrophils) and red (*Shigella*) fluorescence from single or co-infected fish is shown. **(B)** Neutrophil counts in uninfected (CTRL, black symbols) or upon sublethal SINV-GFP (green symbols), *Shigella*-DsRed (red symbols) injection or sequential SINV + *Shigella* (blue symbol) injection. Neutrophils were counted from images taken on live infected larvae using ImageJ software, and plotted as specified in Section “Materials and Methods.” Mean ± SEM are also shown (horizontal bars). Data plotted are from two pooled independent experiments (*n* = 7 larvae scored for each condition).

First, we assessed the population of neutrophils at the whole body-level, using reporter transgenic zebrafish larvae harboring green neutrophils Tg(*mpx*:GFP)*^i114^* (28), referred herein as *mpx*:GFP. While SINV-infected cells also expressed GFP, the fluorescence of individual *mpx*:GFP^+^ neutrophils was much stronger, the only exception being some dense clusters of SINV-infected cells in the brain, an organ which is devoid of neutrophils. Therefore, the identity of neutrophils under the fluorescence microscope was unambiguous (Figure 3A). We counted neutrophils from images of SINV + *Shigella* infected larvae, which we compared with uninfected larvae and larvae inoculated with SINV only at 72 hpf or *Shigella* only at 96 dpf (Figure 3B). Images were taken at 100 and 120 hpf, corresponding to 4 and 24 h after *Shigella* inoculation, respectively. As expected, in *Shigella* only infected larvae, neutrophil numbers did not change significantly, consistently with neutrophil quantification following sublethal *Shigella* inoculation at 72 hpf (27). Interesting, in SINV only infected animals, a significant increase of neutrophils was observed at 120 hpf, similarly to what had been described previously with CHIKV infection (25). Strikingly, neutrophil numbers decreased in SINV + *Shigella* co-infected larvae as soon as 4 h after *Shigella* infection (*p* < 0.05), and this reduction was even more pronounced the following day (*p* < 0.001).

Collectively, these observations show that a significant fraction of neutrophils undergo cell death *in vivo* when *Shigella* infection is preceded by SINV infection.

### Impaired Neutrophil Recruitment and Survival in SINV + Local *Shigella* Co-Infection

To better observe the fate of neutrophils during SINV + *Shigella* co-infection, we replaced the bloodstream inoculation of *Shigella* by a subcutaneous inoculation in mid-trunk. The bacterial infection is thus essentially limited to the flat, thin space between the epidermis and 2 or 3 chevron-shaped somites, allowing detailed time-lapse imaging of phagocyte recruitment and of cell–cell and cell–bacteria interactions by confocal fluorescence microscopy (35).

**Figure 4:**
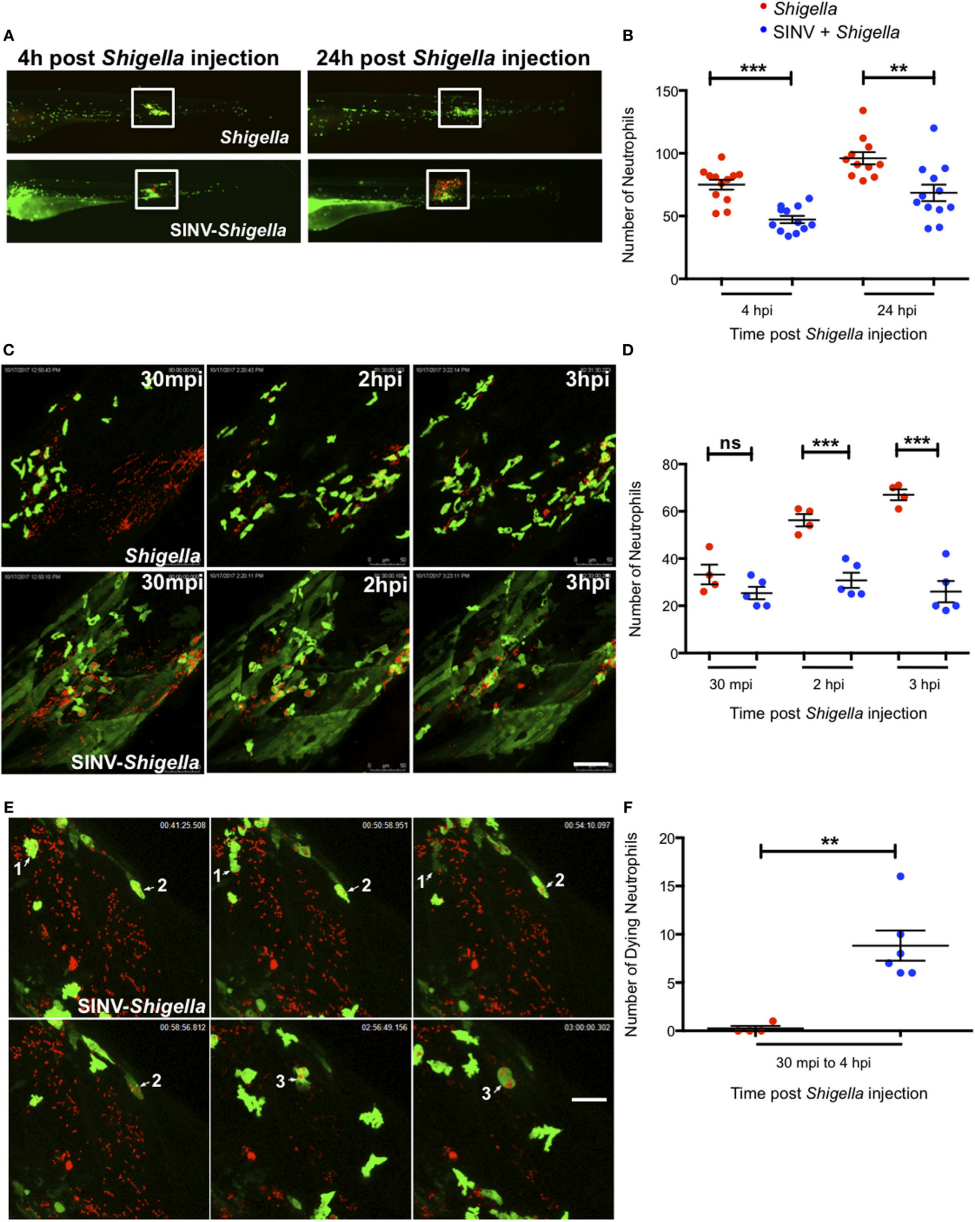
Impaired neutrophil recruitment and survival in Sindbis virus (SINV)- > local *Shigella* co-infection. **(A)** 72 hpf *mpx*:GFP larvae were sequentially injected with SINV-GFP in the bloodstream then subcutaneously with *Shigella*-DsRed one day later (96 hpf). As control, *mpx*:GFP larvae were injected subcutaneously with *Shigella* only at 96 hpf. The infected larvae were imaged with a fluorescent stereomicroscope over time at 100 hpf (4 h post *Shigella* infection) and at 120 hpf (24 h post *Shigella* infection), to monitor neutrophil recruitment to the locally injected bacteria. Overlay of green (SINV and neutrophil) and red (*Shigella*) fluorescence is shown. The white box indicates the region chosen to count the recruited neutrophils. **(B)** Neutrophil recruitment quantification upon sublethal *Shigella*-DsRed (red symbol) injection or sequential SINV + *Shigella* (blue symbols) injection. Neutrophils were counted from images taken on live infected larvae [white box delimitated the region chosen to count the recruited neutrophils in **(A)**] using ImageJ software, and plotted as specified in Section “Materials and Methods.” Data are from one experiment (*n* = 12 larvae scored for each condition). Mean ± SEM are also shown (horizontal bars). **(C)** Frames extracted from maximum intensity projection of *in vivo* time-lapse confocal imaging sessions of 96 hpf *mpx*:GFP larvae injected subcutaneously with *Shigella*-DsRed alone (top panel) or of SINV + *Shigella* co-infected larvae that had been injected one day before with SINV-GFP in the bloodstream (at 72 hpf) (bottom panel). Overlay of green (SINV and neutrophils) and red (*Shigella*) fluorescence of the caudal area of the larvae is shown. Time indicated on the frames is upon subcutaneously *Shigella* injection. See also Video S1 in Supplementary Material. Scale bar: 50 µm. **(D)** Neutrophil recruitment quantification upon subcutaneous *Shigella*-DsRed (red symbol) injection or sequential bloodstream SINV-GFP injection followed the day after by subcutaneous *Shigella-*DsRed (blue symbols) injection. Neutrophils were manually counted at 30 min, 2 and 3 h post *Shigella* injection from maximum intensity projections frames of confocal acquisitions of live infected larvae (to count the recruited neutrophils the region taken into consideration is shown in **(B)** and plotted as specified in Section “Materials and Methods.” Data plotted are from *n* = 4 to 5 larvae scored for each condition. Mean ± SEM are also shown (horizontal bars). **(E)** Frames extracted from maximum intensity projection of confocal acquisition of SINV + *Shigella mpx*:GFP co-infected larvae. SINV-GFP was injected in the bloodstream at 72 hpf and *Shigella*-DsRed was subcutaneously injected the day after, at 96 hpf. The acquisition of the infected larvae was started about 30 min after *Shigella* injection. Three dying *Shigella* engulfing neutrophils are shown (annotated as 1, 2, and 3 on the frames). Overlay of green (SINV and neutrophils) and red (*Shigella*) fluorescence of the caudal area of the larvae is shown. Time indicated on the frames is upon subcutaneously *Shigella* injection. See also Video S2 in Supplementary Material. Scale bar: 20 µm. **(F)** Dying neutrophils quantitation upon subcutaneous *Shigella*-DsRed (red symbol) injection or sequential bloodstream SINV-GFP injection followed the day after by subcutaneous *Shigella-*DsRed (blue symbols) injection. Dying neutrophils were manually tracked and quantified from maximum intensity projections of confocal acquisitions and plotted as specified in Section “Materials and Methods.” Data plotted are from *n* = 4 *Shigella*-infected larvae and *n* = 6 SINV + *Shigella*-infected larvae scored. Mean ± SEM are also shown (horizontal bars).

We thus addressed the impact of SINV + *Shigella* co-infection on the ability of neutrophils to sense, migrate, and be recruited toward a local *Shigella* inoculum. We injected a sub lethal GFP-SINV inoculum in the bloodstream of 72 hpf *mpx*:GFP larvae, and the day after we injected about 2 × 10^3^ *Shigella*-DsRed subcutaneously (a sublethal dose also by this route; not shown); as a control, we injected the same *Shigella* inoculum in previously uninfected fish (Figure 4). We first quantified neutrophil recruitment to the bacteria at 100 and 120 hpf, corresponding to 4 and 24 h post *Shigella* injection, using a wide field fluorescent microscope (Figures 4A,B). As expected, many neutrophils were already recruited by 4 h post *Shigella* injection, with co-localization of green and red fluorescence suggesting that neutrophils had started to engulf bacteria. The recruited neutrophils were still there 24 h post *Shigella* injection, and their numbers slightly increased, presumably due to bacterial invasion and proliferation in muscle fibers that die sporadically, releasing live bacteria quickly engulfed by neutrophils as we previously showed (27). Although recruitment occurred in both single- and co-infected larvae, the local neutrophil population was significantly decreased in the co-infected larvae at both time points (Figure 4B), indicating that the previous viral infection resulted in deficient recruitment and/or survival of neutrophils to the bacterial site.

To analyze the *Shigella*–neutrophil interactions in more detail, we recorded neutrophil behavior by live imaging at high resolution using a confocal microscope, documenting the early steps of neutrophil recruitment, between 30 min and 3 h post subcutaneous *Shigella* injection (Figure 4C; Video S1 in Supplementary Material). At the beginning of the acquisition, we found neutrophils already recruited to the bacteria, in comparable numbers in SINV + *Shigella* and *Shigella*-only injected animals. As expected, the number of recruited neutrophils progressively increased in *Shigella*-only injected larvae. However, the scenario was very different in the SINV + *Shigella*-injected animals, where the number on neutrophils did not increase, thus becoming significantly lower than in controls from 2 h post *Shigella* injection (Figure 4D).

Closer examination of the time-lapse movies revealed that these recruited neutrophils could undergo cell death upon *Shigella* engulfment in SINV + *Shigella*-injected animals (Figure 4E; Video S2 in Supplementary Material), something not previously observed in *Shigella*-only infected larvae. We thus quantified the number of neutrophils dying upon having engulfed the bacteria from 30 min to 4 h post *Shigella* injection by manually tracking neutrophils on live imaging acquisitions. This quantification confirmed that a significant number of *Shigella*-containing neutrophils died in co-infected fish, while none did in *Shigella*-only infected controls (Figure 4F; Videos S1 and S2 in Supplementary Material).

Collectively, these observations demonstrate that neutrophil anti-bacterial functions are perturbed in SINV + *Shigella* co-infected animals: neutrophil recruitment toward the bacteria is impaired, and phagocytosing neutrophils undergo cell death. Overall, they strongly suggest that the viral response initiated upon SINV injection interferes with the bacterial response initiated upon *Shigella* injection, resulting in uncontrolled *Shigella* proliferation and dissemination in co-infected fish.

### Immune Gene Modulation Upon SINV + *Shigella* Co-Infection

**Figure 5:**
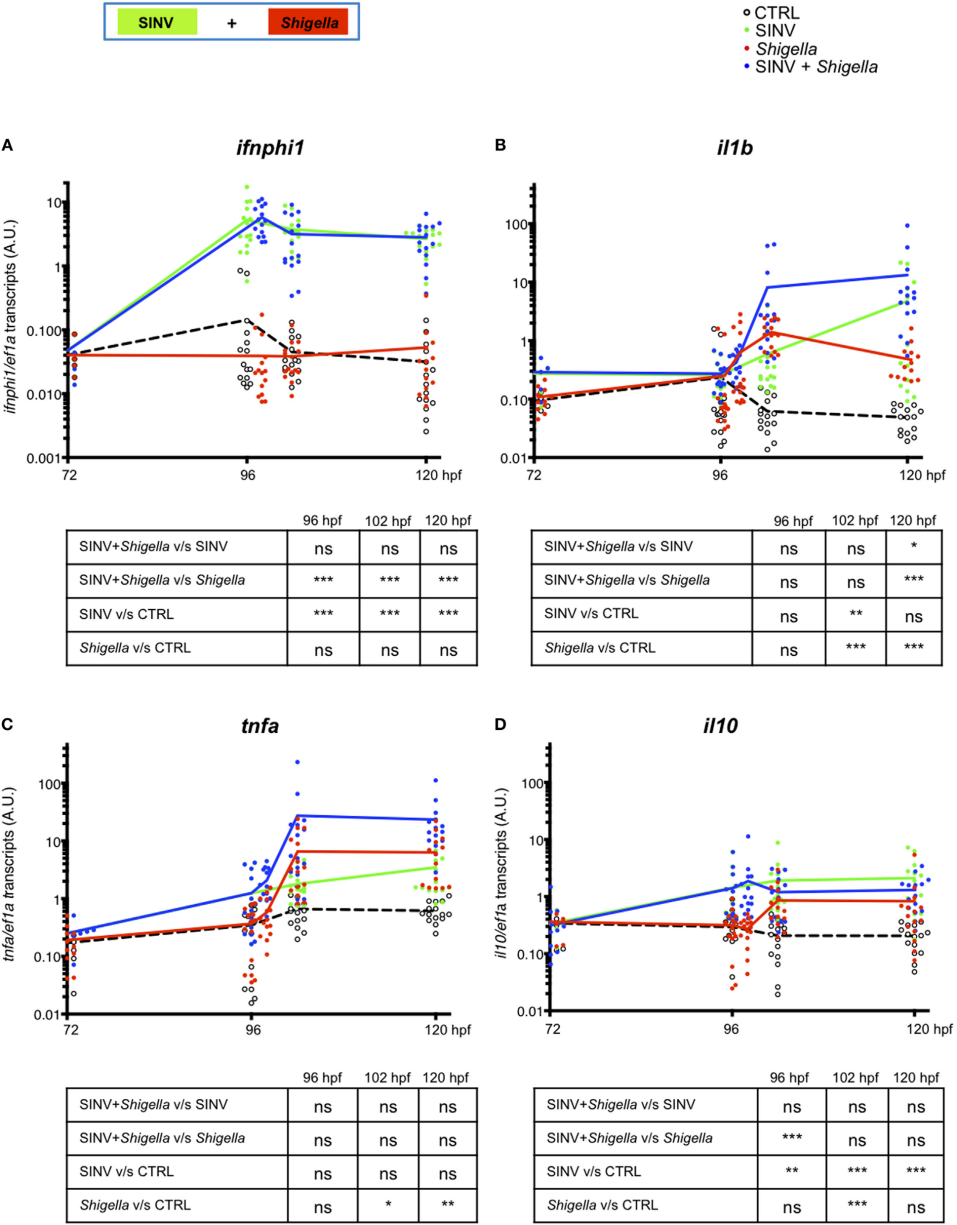
Cytokine gene modulation upon Sindbis virus (SINV) + *Shigella* co-infection. **(A–D)** Cytokine (*ifnphi1, il1b, tnfa, il10*) induction was measured from individual zebrafish larvae sequentially co-injected with SINV + *Shigella* (blue) or from individual zebrafish larvae injected with SINV alone (green) or with *Shigella* alone (red), and non-injected fish as control (CTRL, black curves). Data plotted are from three independent experiments pooled (*n* = 15 larvae for each condition); individual values are shown and curves correspond to the means. Statistical analysis is shown as a table under each graph.

Finally, we measured cytokine gene expression by qRT-PCR in SINV + *Shigella* and corresponding single-infected fish. We first measured *ifnphi1* and *il1b*, two signature cytokines of anti-viral and anti-bacterial responses, respectively. As expected, a strong and sustained type I IFN response was detected in SINV-only infected fish, while *Shigella* only did not induce any detectable IFN induction (Figure 5A). IFN expression in co-infected fish was strictly similar to that of SINV-only infected fish, consistent with the fact that SINV burden is not affected by bacterial superinfection (Figures 2E,F). Reciprocally, a strong *il1b* response was rapidly induced in *Shigella*-only infected fish; SINV induced its expression much more slowly. However, while this response was transient in *Shigella*-only infected larvae, it was sustained in SINV + *Shigella* co-infected animal (Figure 5B). Of note, in *Shigella* + SINV co-infections, *ifnphi1* and *il1b* induction corresponded to the addition of those induced by single infections, again fitting with the absence of interference of the two responses when the two pathogens were administrated in that order (Figures S4A,B in Supplementary Material).

This increased expression of *il1b* in SINV + *Shigella* co-infections is mostly seen in late (24 h) but not early (6 h) time after *Shigella* injection, paralleling the increased bacterial burden of these animals (Figure 2C), making it unclear whether it is a cause or a consequence of higher bacterial loads. Since deficiency of neutrophil function is already observed a few hours after *Shigella* injection (Figures 3 and 4), we tested the expression of several other candidate genes at 6 h post *Shigella* injection (Figure S3 in Supplementary Material). Genes typically associated with bacterial, but not viral infection, such as *il8* (*cxcl8a*), *tnfa, il22*, were indeed not induced by SINV alone but were induced by *Shigella*, while *mmp9* was also induced by SINV, as previously observed with CHIKV (39). For other genes tested, no obvious interaction was revealed, as expression in dually infected fish was comparable to that of SINV or *Shigella* single-infected fish. Interestingly, the anti-inflammatory cytokine *il10* was induced by SINV only at this time point (6 h post *Shigella* injection). Thus, from these observations, we decided to measure the kinetics of induction of *tnfa* and *il10* over time comparing SINV or *Shigella* single injected fish to SINV + *Shigella* co-infected fish (Figures 5C,D). The induction of *tnfa* paralleled the induction of *il1b*, as expected (5 C). Strikingly, we found that *il10* was strongly induced by SINV only by 96 hpf, just before *Shigella* injection, and remained high over time in both SINV and SINV + *Shigella* co-injected fish (Figure 5D). We measured the induction of these genes in *Shigella* + SINV co-infected fish, showing no obvious interference between the antiviral and antibacterial induced genes when *Shigella* was injected first (Figures S4C,D in Supplementary Material). We also measured the kinetics of SINV dependent-*mmp9* (encoding for Matrix metalloproteinase 9) induction upon SINV + *Shigella* or *Shigella* + SINV co-infections, showing that *mmp9* was strongly induced only when SINV was injected before *Shigella*, again suggesting an interference between the antiviral and the antibacterial induced genes only when virus is injected first (Figures S5A,B in Supplementary Material). We also tested possible correlation of cytokine expression and bacterial burden (asses by qRT-PCR) at 120 hpf, to see if differences could be observed between controller and no-controllers SINV + *Shigella* co-infected fish. As reported on Figure S6 in Supplementary Material, no obvious correlation between bacterial burden and cytokine expression was observed, except for *il1b*, which is correlated with burden in co-infected but not in *Shigella*-only infected fish.

Overall, these observations suggest that, in addition to type I IFN, the SINV-dependent *il10* induction measured in SINV + *Shigella* co-infected fish, given the anti-inflammatory properties of this cytokine, could participate in the increased susceptibility to bacterial infection with the concomitant death and uncontrolled bacterial proliferation observed in SINV + *Shigella* co-infected fish.

## Discussion

Hyper-susceptibility to secondary bacterial infection following acute viral infections is a major clinical issue, for which animal models are indispensable to understand the underlying mechanisms and test therapeutic and prophylactic approaches (6). In that respect, mouse models have yielded remarkable insights, yet alternative models could also provide complementary information and valuable tools. The optically and genetically tractable swimming zebrafish larva constitutes a particularly attractive system. Here, using two infection models previously developed by our team with SINV (24) and *Shigella* (27), we describe the first instance of virus-induced bacterial hyper-susceptibility in zebrafish, and show that this susceptibility is associated with virus-induced defects in neutrophil function. Of note, another polymicrobial infection model, combining yeast and bacteria, has also been recently described in zebrafish (40).

To our knowledge, SINV and *Shigella* are not associated in co-infections in humans. This possibility is not excluded, since SINV infection has been largely neglected in humans, as it is considered to be mild (41). Interestingly, an outbreak of influenza virus H1N1 and *Shigella flexneri* co-infection was reported in a precarious and overcrowded gold miner camp in the tropical forest of French Guiana (42).

We do not think that the increased susceptibility to bacterial infection we observed in zebrafish larva upon SINV infection is specific to *Shigella flexneri*. We consider our model as a tool to address the possible interference of well-defined canonical anti-viral and anti-bacterial responses *in vivo*, beyond the specificities of SINV and *Shigella*. Other virus–bacterium combinations will be tested in zebrafish in the future to test this hypothesis.

This zebrafish viral–bacterial co-infection model offers great practical advantages. First, the timeframe of the experiments is quite short: less than a week from crossing breeding adults to final results. Second, microbe injections are performed at 72 and 96 hpf, late enough for the innate immune system of the larva to be operative, and yet early enough for use of many transient genetic manipulation approaches such as morpholino-mediated knockdown. Finally, the transparency, small size, and easy anesthesia of the zebrafish larva makes it quite easy to monitor the extant and spread of infections over time, and the combination of two different reporter fluorescent SINV and *Shigella* strains allow simultaneous observation of virus-infected cells and of bacteria dissemination. SINV and *Shigella* are both BSL2 pathogens, with many well-established genetic tools, and many other fluorescent colors are available beyond the GFP and DsRed used in this report. Thus, the various fluorescent zebrafish lines available, reporting immune cells or cytokine responses (25, 43, 44), can be combined in diverse ways with the fluorescent microbes, allowing the monitoring of the orchestration of the innate immune response and microbe-immune cell interactions in real time at the scale of the entire organism.

Timing is a key parameter when superinfection models are considered. In murine influenza-based models, hyper-susceptibility to bacteria is observed if 7 days elapse between the two infections, but not with a 3-day delay (16). In the zebrafish larva co-infection model described here, a 24-h time lapse between the two microbes was sufficient to detect a robust hyper-susceptibility to bacteria in virus infected animals. It would be worthwhile to more precisely determine the hyper-susceptibility time window in the zebrafish co-infection model in the future, by testing a range of delays, including simultaneous inoculation. Dosage of either microbe, predictably, is another key parameter, and we had to perform many tests (Table S1 in Supplementary Material) before finding the experimental conditions reported here.

Interestingly, while we found that virus-infected larvae were hyper-susceptible to bacteria, infecting with bacteria first and virus later did not result in increased mortality. Although one should certainly not derive any general conclusion from this observation, this appears remarkably similar to what has been observed in mouse models (45), and perhaps in humans as well, as hyper-susceptibility to viruses is not a notorious issue in bacteria-infected patients. The origin of this asymmetry would be worth investigating.

What are the molecular mechanisms that underlying the hypersensitivity we report here? Are they similar to those described in mice? Clearly, this will be our next line of investigation. The role of the type I IFN response, well-established in mouse, would be the first to address. Unfortunately, knocking down type I IFN receptor chains in zebrafish larvae results in death from the SINV dose used here (Figure S1 in Supplementary Material), requiring alternative approaches, such as injection of recombinant zebrafish type I IFNs, which will require extensive tests of IFN subtype, dosage, and timing.

Quite possibly, only one or a few of the hundreds of genes—mostly IFN-stimulated genes (ISGs)—induced by SINV infection could underlie the phenotype. In this context, it has been recently reported in a mouse model of influenza virus and *Streptococcus pneumoniae* co-infection, that the IFN-inducible methyltransferase Setdb2 mediates virus-induced susceptibility to bacterial infection, perturbing neutrophil functions by repressing the expression of genes encoding neutrophil attractant mediators like CXCL1 and other genes that are targets of the transcription factor NF-kB. Thus, Setdb2 could mediate the regulation of type I IFN and NF-kB pathways cross talk and could represent one of the mechanisms involved in virus induced susceptibility to bacterial superinfections (46). IFNs may even modify the phenotype of neutrophils independent of ISG induction, as recently shown for type III IFN and reactive oxygen species production (47).

We have shown that *il10* is induced upon SINV infection, and that its level remains high when *Shigella* is injected. Because of its known anti-inflammatory properties, IL-10 could be responsible of the increased bacterial susceptibility of viral infected fish, by impairing phagocyte anti-bacterial functions. In this context, it has been shown that IL-10 impairs neutrophil recruitment to infected tissues in a neonatal mouse model of bacterial sepsis, and that perturbing IL10 induction resulted in the rescue of efficient neutrophil recruitment, bacterial clearance, and increased survival (48). Moreover, IL-10 expression prior to bacterial infection was shown to inhibit neutrophil recruitment, resulting in insufficient bacterial clearance and increased mortality in a mouse model of pneumonia (49). IL10 is thus an obvious candidate to test in our co-infection model.

We addressed the possible role of neutrophils in the hypersusceptibility phenotype and found that in co-infected animals, neutrophils frequently died after having engulfed bacteria. We cannot exclude that the death of other cell types also contribute to the phenotype, the most likely candidates being macrophages. However, as we have previously documented that, unlike neutrophils, some *Shigella*-infected macrophages already undergo cell death upon low dose *Shigella* infection (2,000 CFU, used in this study) (27), and therefore, we decided to focus on neutrophil behavior only. Interestingly, while found that even though viral infection increases the total neutrophil population (Figure 3), it also makes these cells less able to cope with bacteria. Intriguingly, in zebrafish, neutrophils themselves can be an important source of type I IFN (25), suggesting differentiation into virus-targeted cells to the detriment of their antibacterial function. IFN-dependent polarization of neutrophils into distinct “N1” and “N2” phenotypes has been proposed as an important mechanism in tumor rejection (50). In this context, our SINV-*Shigella* co infection model will allow to address if type I IFN-producing neutrophils are still able to sense, migrate to and engulf bacteria, or if they are a specialized neutrophil subset that have lost their antibacterial functions.

Expression of pro-inflammatory cytokine genes such as *il1b* and *tnfa* is rapidly induced by many bacterial infections, and *Shigella* is no exception (Figure 5; Figure S4 in Supplementary Material). These transcripts are more strongly upregulated in SINV + *Shigella* compared to *Shigella* only infected fish. These cytokines normally contribute to antibacterial defense, and this higher expression may be just a consequence of the higher bacterial burden of these animals, without leading to the hypersusceptibility phenotype. However, a causal (deleterious) role cannot be ruled out, perhaps linked to pyroptosis-mediated demise of myeloid cells. Pyroptosis of macrophages has been observed in zebrafish larvae infected with SVCV virus (51); however, the situation is quite different here as (i) unlike SINV, SVCV is a very poor IFN inducer (52) and (ii) unlike SVCV, SINV does not infect macrophages (24). A more detailed study of what cells express these cytokines during the co-infection using appropriate reporters (43, 44), and if they undergo inflammasome oligomerization (53), should illuminate this issue. To note, using a zebrafish model of local *Shigella* infection to perturb the cytoskeletal septins proteins expression, we recently reported deregulated inflammatory response and neutropenia and showed that too much IL1β-dependent inflammation resulted in the increased susceptibility of neutrophils to *Shigella* infection, with concomitant death of the engulfing neutrophils (36). It will be interesting to check if Anakinra, a IL1β receptor antagonist, that we have shown to rescue neutrophil death and host survival upon *Shigella* infection in septin depleted fish in this model (36), could also be able to rescue neutrophil functions and host survival in the SINV-*Shigella* co infection model we described here.

Although we still do not know if the mechanisms that lead to increased bacterial susceptibility upon a viral infection are shared by fish and mammals, the evolutionary conservation of this phenomenon is in itself remarkable. Since it is obviously counter-adaptive in some situations, one may infer that the immune modulation induced by the antiviral response provides a significant fitness advantage overall. This situation fits with the general concept of “immunity by equilibrium” (54), even if the aforementioned asymmetry of the virus-then-bacteria and bacteria-then-virus situations remain to be explained.

In conclusion, we describe here a new model of sequential infection of zebrafish larva with a virus (SINV) and a bacterium (*Shigella*), that uncovers the conservation of the virus-induced hyper-susceptibility to bacterial superinfection in this host. This opens up numerous avenues to unravel the mechanisms at play in this phenomenon. Importantly, the diminutive zebrafish larva, small enough to fit in microtitration plates, is highly suited to pharmacological screening (55). This system should therefore provide a valuable pre-clinical tool to test new candidate drugs to alleviate secondary bacterial superinfections—therapeutics that would restore the host immune system would be more desirable than current approaches, undermined by mounting antibiotics resistance.

## Ethics Statement

Animal experiments were performed according to European Union guidelines for handling of laboratory animals (http://ec.europa.eu/environment/chemicals/lab_animals/home_en.htm) and were approved by the Institut Pasteur Animal Care and Use Committee.

## Author Contributions

LB generated most images and qPCR results, analyzed data, and made the figures. GP carried out most viral infections. VT analyzed the first co-infections experiments. LM performed infections in morphant larvae. PH contributed to funding acquisition. JPL and ECG conceived the study, performed infections, supervised the work, analyzed the data, and wrote the manuscript.

## Conflict of Interest Statement

The authors declare that the research was conducted in the absence of any commercial or financial relationships that could be construed as a potential conflict of interest. The reviewer SM declared a past co-authorship with the authors to the handling Editor.

## Supplementary Material

Figure S1 Dependence on type I interferons (IFN) response for survival to Sindbis virus (SINV) infection. Zebrafish embryos were injected with CRFB1 and CRFB2-specific morpholinos at the 1-cell stage to generate larvae deficient in type I IFN receptors, or with control morpholinos. At 72 hpf, they were infected with ~60 PFU of SINV-GFP2A (*n* = 8–10 per group), and survival was then assessed by daily observation.

Figure S2 Sindbis virus (SINV)-GFP-3′UTR also causes susceptibility to co-infection when injected first. Same experiment and readout than for Figure 2, except that SINV-GFP-3′UTR was used instead of SINV-GFP2A. Two independent experiments pooled. **(A,B)** Survival curves; *n* = 48 fish for each condition. **(C,D)** Bacterial burden quantification; *n* = 8–10 larvae for each condition.

Figure S3 qRT-PCR analysis of cytokine and inflammatory mediators induction, 6 h post *Shigella* injection. Gene expression was measured from individual zebrafish larvae sequentially co-injected with Sindbis virus (SINV) + *Shigella* (blue bars) or from individual zebrafish larvae injected with SINV alone (green bars) or with *Shigella* alone (red bars), and non-injected fish (CTRL, black bars) as control at 102 hpf (corresponding to 6 h post *Shigella* injection). *N* = 5 larvae per condition; note that these data were included among the values plotted on Figure 5.

Figure S4 Cytokine expression upon *Shigella* + Sindbis virus (SINV) co-infection. Same settings and readout as for Figure 5, except that *Shigella* was injected first and SINV second (co-infected fish, cyan symbols). *n* = 5 larvae for each condition in total.

Figure S5 Kinetics of *mmp9* induction upon Sindbis virus (SINV) + *Shigella* and *Shigella* + SINV co-infection. **(A,B)** *mmp9* induction was measured from individual zebrafish larvae sequentially co-injected with SINV-*Shigella* (blue curves) or *Shigella*-SINV (cyan curves), or from individual zebrafish larvae injected with SINV alone (green curves) or with *Shigella* alone (red curves), and non-injected fish (CTRL, black curves) as control.

Figure S6 Possible correlation of cytokine expression levels and bacterial burden in Sindbis virus (SINV) + *Shigella* co-infected larvae. **(A–D)** Cytokine (*ifnphi1, il1b, tnfa, il10*) induction and bacterial content (*DsRed*) was measured from individual zebrafish larvae sequentially co-injected with SINV + *Shigella* (blue) or from individual zebrafish larvae injected with *Shigella* alone (red). Cytokine induction was correlated with bacterial content for each larva. Data plotted are from 3 independent experiments pooled (*n* = 15 larvae for each condition, same larvae showed in Figure 5); individual values are shown.

Video S1 (Related to Figure 4C) Impaired neutrophil recruitment upon Sindbis virus (SINV)-local *Shigella* co-infection. *Mpx*:GFP larvae were injected in the bloodstream with SINV-GFP at 72 hpf and subcutaneously with *Shigella*-DsRed at 96 hpf (panel on the right of the movie), or subcutaneously with *Shigella*-DsRed only at 96 hpf as a control (panel on the left of the movie), and live imaged in the trunk region (where the bacteria were injected) every 2 min from 30 mpi (*t* = 0 on the movie) to 3 h post infection (*t* = 2 h 33 on the movie) simultaneously by confocal fluorescence microscopy. Immediately after the starting of the acquisition, some neutrophils (GFP + bright cells) had already recruited to the bacteria in both conditions. In *Shigella*-only infected larvae (left), more neutrophils were progressively recruited to the bacteria (DsRed+), engulfing them quickly, and accumulating at the site of injection without any sign of cell death. In sequentially SINV + *Shigella*-injected larve (right), neutrophils poorly accumulated at the site of infection, and some underwent cell death upon engulfing bacteria. Note that SINV-GFP (GFP + diffuse signal) had replicated and invaded the muscle fibers of the trunk region. Maximum intensity projection is shown. Scale bar: 50 µm.

Video S2 (Related to Figure 4E) Impaired neutrophil survival upon Sindbis virus (SINV) + *Shigella* co-infection. A 72 hpf *mpx*:GFP larva was injected in the bloodstream with SINV-GFP and at 96 hpf subcutaneously with *Shigella*-DsRed, and live imaged immediately upon *Shigella* injection, every 1′30″ from 30 mpi (*t* = 0 on the movie) to 3 h 39 pi (*t* = 3 h 09 on the movie). Six engulfing neutrophils (GFP + bright cells, indicated by arrows on the movie) were manually tracked over time. Note the SINV-GFP infected cells (diffuse GFP signal, muscle fibers, and mesenchyme in the left bottom corner of the field). Injection site, maximum intensity projection. Scale bar: 20 µm.
