# Supplementary material for: A Model of Superinfection of Virus-Infected Zebrafish Larvae: Increased Susceptibility to Bacteria Associated With Neutrophil Death
Source: Front Immunol. 2018 May 24;9:1084. doi: 10.3389/fimmu.2018.01084 (PMC5976802; doi:10.3389/fimmu.2018.01084)
Supplement: Figure S1 — Dependence on type I interferons (IFN) response for survival to Sindbis virus (SINV) infection. Zebrafish embryos were injected with CRFB1 and CRFB2-specific morpholinos at the 1-cell stage to generate larvae deficient in type I IFN receptors, or with control morpholinos. At 72 hpf, they were infected with ~60 PFU of SINV-GFP2A (n = 8–10 per group), and survival was then assessed by daily observation. [file Data_Sheet_1.PDF]

Figure S1

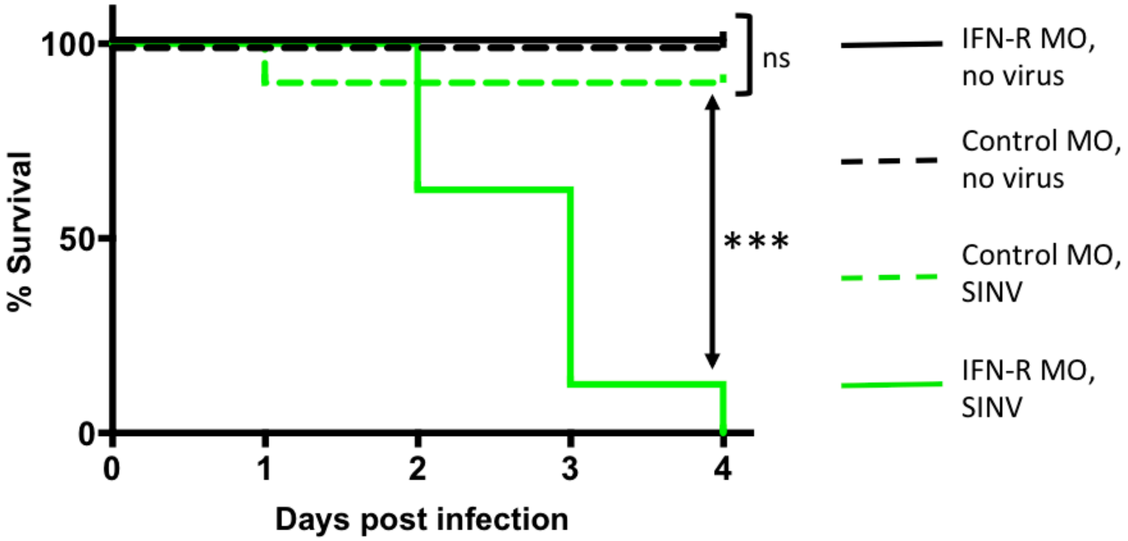

100
50
0
% Survival
0
1
2
3
4
Days post infection
ns
***
IFN-R MO, no virus
Control MO, no virus
Control MO, SINV
IFN-R MO, SINV

Figure S2

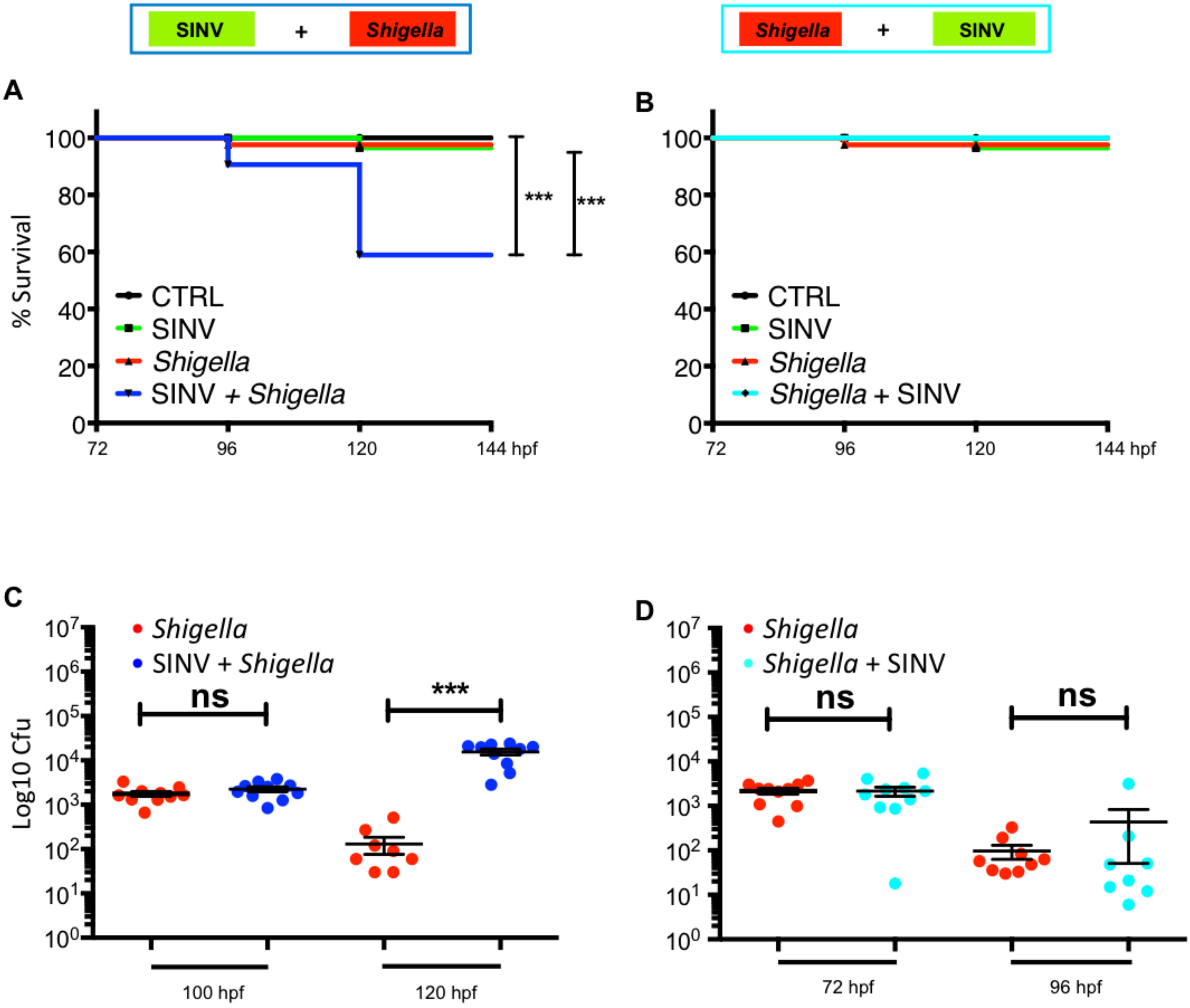

SINV + Shigella
Shigella + SINV
A
B
% Survival
100
80
60
40
20
0
72
96
120
144 hpf
***
***
CTRL
SINV
Shigella
SINV + Shigella
Shigella + SINV
C
D
Log10 Cfu
ns
***
100 hpf
120 hpf
72 hpf
96 hpf

Figure S3

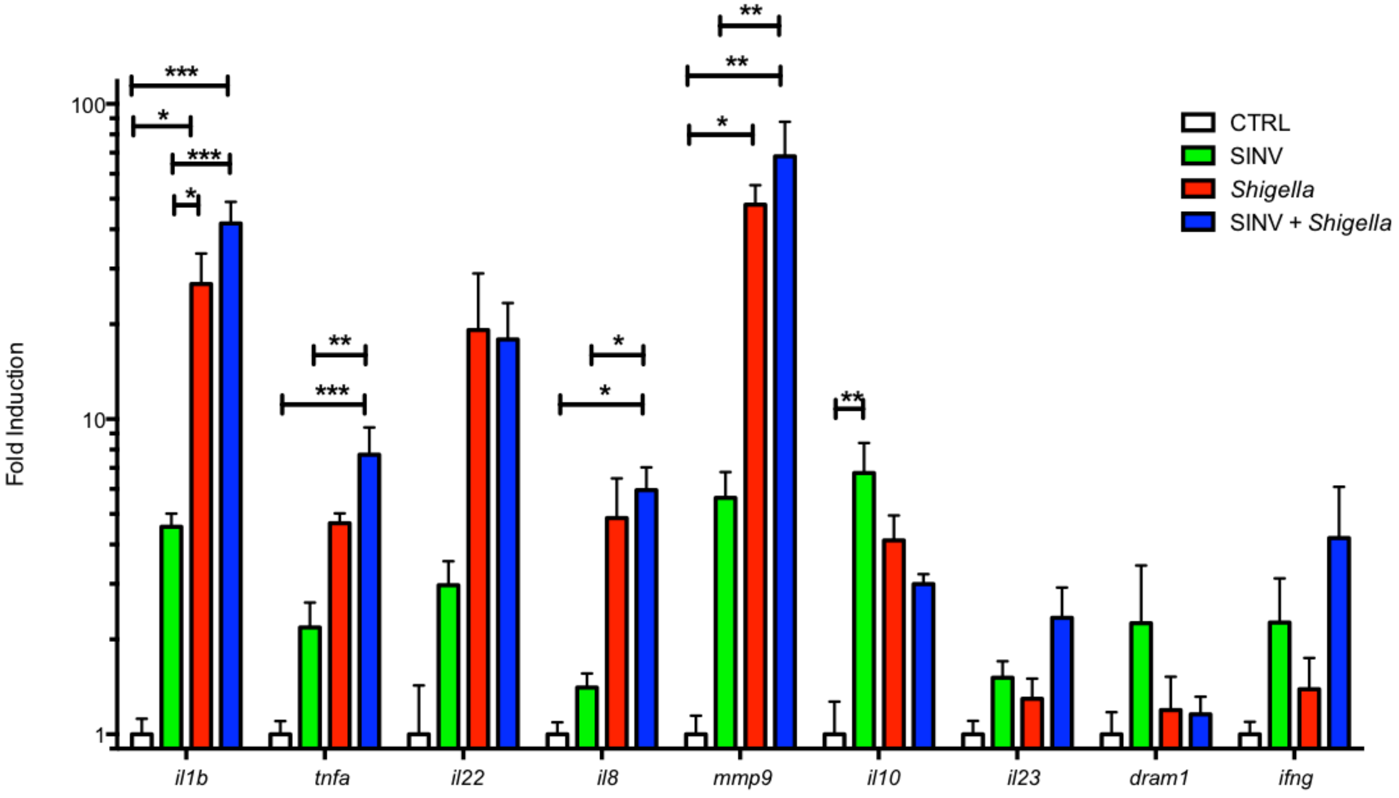

***
*
***
*
**
***
*
*
**
**
*
**
100
10
1
Fold Induction
CTRL
SINV
Shigella
SINV + Shigella
il1b
tnfa
il22
il8
mmp9
il10
il23
dram1
ifng

Figure S4

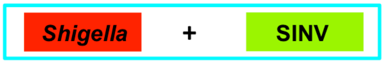

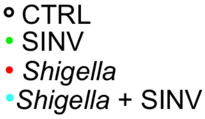

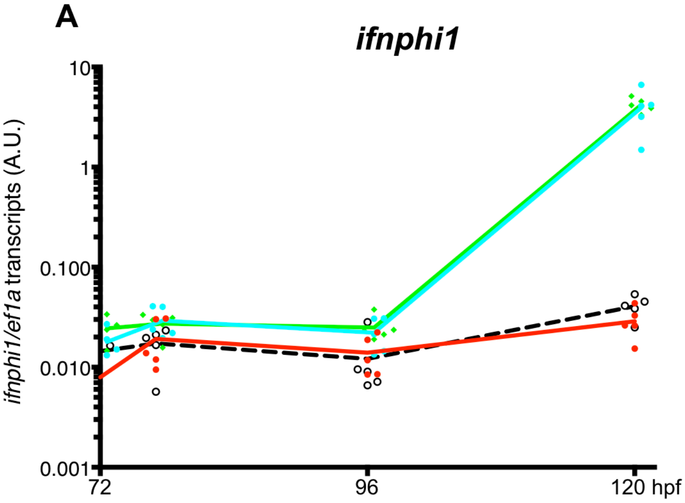

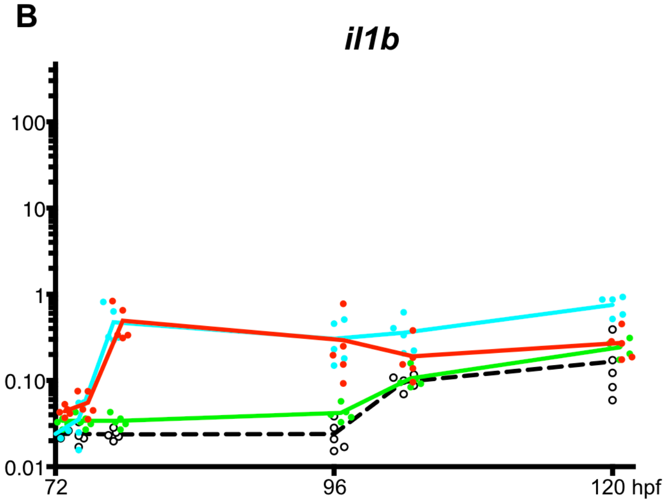

	78 hpf	96 hpf	120 hpf
Shigella+SINV v/s SINV	ns	ns	ns
Shigella+SINV v/s *Shigella*	ns	ns	***
SINV v/s CTRL	ns	ns	***
Shigella v/s CTRL	ns	ns	ns

	78 hpf	96 hpf	120 hpf
Shigella+SINV v/s SINV	***	**	***
Shigella+SINV v/s *Shigella*	ns	ns	***
SINV v/s CTRL	ns	ns	ns
Shigella v/s CTRL	***	*	ns

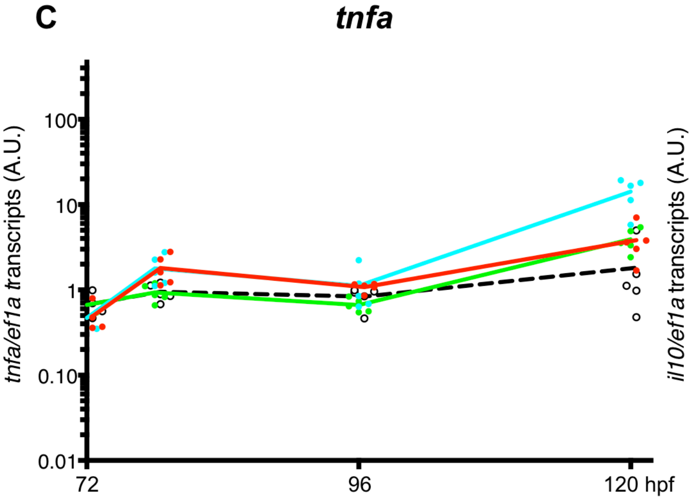

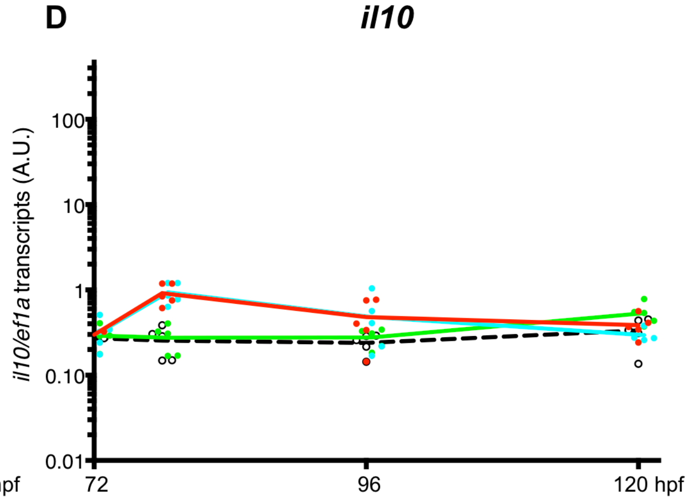

	78 hpf	96 hpf	120 hpf
Shigella+SINV v/s SINV	ns	ns	***
Shigella+SINV v/s *Shigella*	ns	ns	***
SINV v/s CTRL	ns	ns	ns
Shigella v/s CTRL	ns	ns	ns

	78 hpf	96 hpf	120 hpf
Shigella+SINV v/s SINV	***	ns	ns
Shigella+SINV v/s *Shigella*	ns	ns	ns
SINV v/s CTRL	ns	ns	ns
Shigella v/s CTRL	***	ns	ns

Figure S5

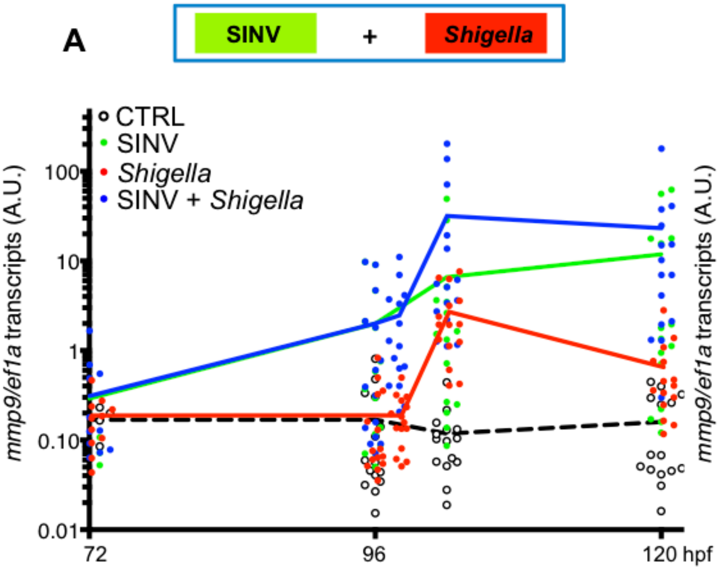

	96 hpf	102hpf	120 hpf
SINV+*Shigella* v/s SINV	ns	ns	ns
SINV+*Shigella* v/s *Shigella*	ns	ns	*
SINV v/s CTRL	ns	**	***
Shigella v/s CTRL	ns	***	ns

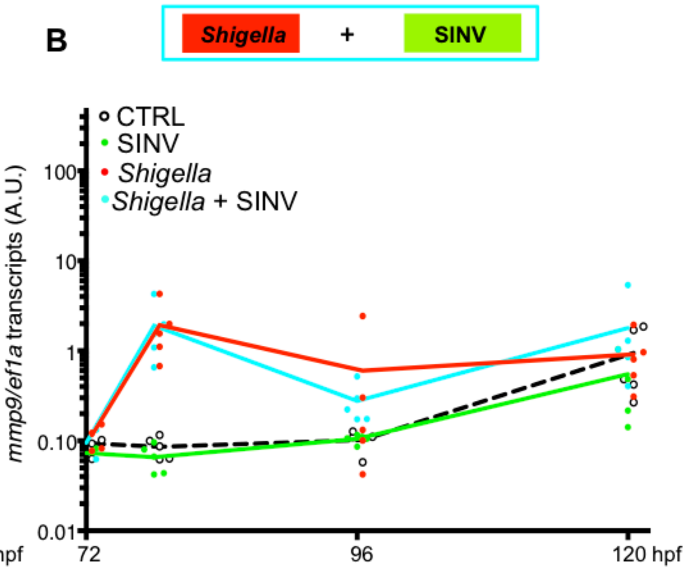

	78 hpf	96 hpf	120 hpf
Shigella+SINV v/s SINV	**	ns	ns
Shigella+SINV v/s *Shigella*	ns	ns	ns
SINV v/s CTRL	ns	ns	ns
Shigella v/s CTRL	**	ns	ns

Figure S6

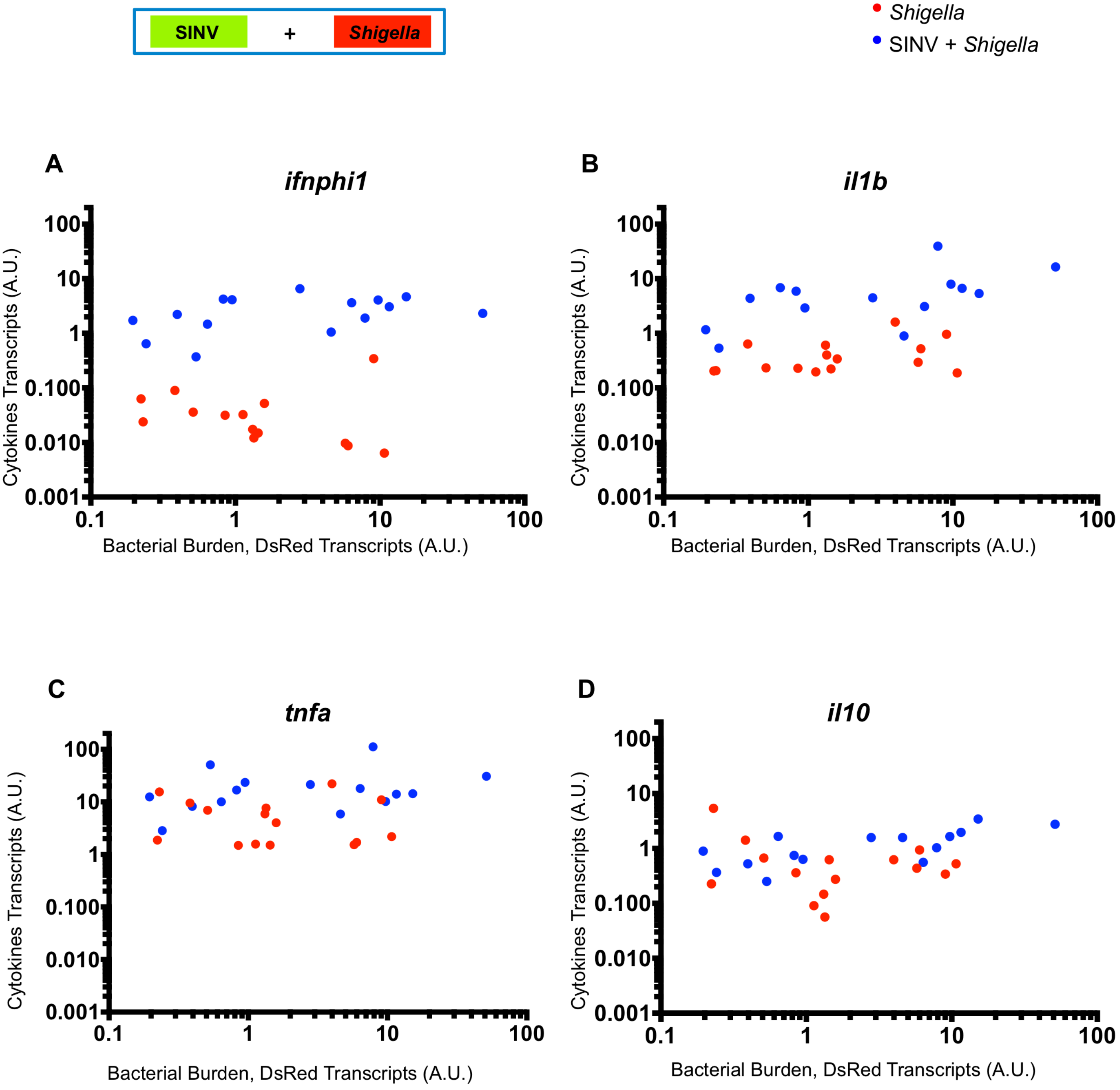
SINV
+
Shigella
Shigella
SINV + Shigella
A
ifnphi1
B
il1b
C
tnfa
D
il10
Cytokines Transcripts (A.U.)
Bacterial Burden, DsRed Transcripts (A.U.)
0.001
0.010
0.100
1
10
100
0.1
1
10
100

Table S1

Listing of conditions tested for the dosage of either microbes

SINV dose	*Shigella* dose	SINV Survival	*Shigella* Survival	SIN+*Shigella* Survival
30 PFU	600 CFU	100%	100%	100%
60 PFU	700 CFU	100%	100%	90%
60 PFU	1500 CFU	100%	95%	50%
100 PFU	1800 CFU	80%	95%	40%
100 PFU	2000 CFU	95%	95%	55%
